# Navigating Complexities: A Case of Multiple Abdominopelvic Vascular Compression Syndromes in Ehlers-Danlos Syndrome

**DOI:** 10.7759/cureus.63848

**Published:** 2024-07-04

**Authors:** Ahmad Mohammed, Samrawit W Zinabu, Miriam B Michael

**Affiliations:** 1 Internal Medicine, Howard University Hospital, Washington, D.C., USA; 2 Internal Medicine, University of Maryland, Baltimore, USA

**Keywords:** superior mesenteric artery syndrome, median arcuate ligament syndrome, may-thurner, nutcracker, ehlers-danlos syndrome, vascular compression syndromes

## Abstract

Abdominopelvic vascular compression syndromes (VCS) refer to conditions where blood vessels in the abdomen or pelvis are compressed by nearby structures, leading to various symptoms and complications. These conditions include superior mesenteric artery syndrome (SMAS), nutcracker syndrome (NCS), May-Thurner syndrome (MTS), and median arcuate ligament syndrome (MALS). Each syndrome is characterized by specific compressions of blood vessels, resulting in symptoms such as pain, nausea, vomiting, weight loss, leg swelling, and other related issues. Ehlers-Danlos syndrome (EDS), characterized by hyperelasticity, altered collagen, and mobility of the viscera, has been associated with VCS, although the exact prevalence is unknown. We report a case of a patient with EDS who presented with multiple VCS, including NCS, MTS, SMAS, and MALS.

## Introduction

Vascular compression syndromes (VCS) are a group of rare but significant medical conditions characterized by compression of blood vessels within the abdominal and pelvic cavities. These syndromes can lead to a variety of symptoms, such as chronic pain, gastrointestinal disturbances, and blood flow-related issues causing aneurysms, thromboses, and emboli [1]. The primary syndromes include median arcuate ligament syndrome (MALS), nutcracker syndrome (NCS), superior mesenteric artery syndrome (SMAS), and May-Thurner syndrome (MTS). Each syndrome is associated with specific vascular structures and clinical manifestations. Understanding these syndromes is critical for timely diagnosis and appropriate management, enabling patients to achieve better outcomes and an improved quality of life.

Ehlers-Danlos syndrome (EDS) is a group of rare, inherited connective tissue disorders characterized by abnormal collagen synthesis and affecting primarily the skin, joints, and blood vessel walls; its frequency among the general population is 1 in 5,000 [2]. The inherited abnormalities involve alterations in genes vital to the synthesis and processing of different types of collagen that are fundamental to the structure and function of various kinds of tissues in the body [2]. This defective synthesis often causes patients to present with a wide assortment of symptoms ranging from mild to life-threatening, such as hyperextensibility of the skin, tissue fragility, vascular tissue dysfunction, and hypermobility of the ligament, which is the most classical manifestation of the condition [3]. According to the latest international classification accepted in 2017, 13 variants of EDS are further classified into six types, the most common being hypermobility and vascular type, each associated with unique genetic collagen mutations and clinical features [2-4]. As such, there can be significant overlap with patients presenting with both EDS and VCS.

## Case presentation

A 26-year-old woman with a medical history of EDS, mast cell activation, and Chiari malformation along with tethered cord syndrome (status post-occipital decompressions and sacral laminectomy with cord untethering) and nonepileptic seizures with multiple negative electroencephalogram (EEG) results.

During a recent visit to the University of Maryland Hospital Emergency Room following a syncopal episode, she was given 4 mg of lorazepam intravenously administered slowly for suspected seizure activity. She also had a drop in blood pressure and subsequent bradycardia, which improved after receiving IV fluids. Before this episode, she had severe abdominal pain linked to eating, leading to a significant weight loss. The patient also reported palpitations, difficulties in concentration due to mental clouding, and dizziness upon standing. Additionally, she complained of left leg swelling, pelvic pain unrelated to menstruation, and intermittent hematuria. On physical examination, the patient was cachectic and had multiple areas of ecchymosis. Her vital signs indicated a heart rate of 52 beats per minute (BPM), a blood pressure of 85/46 mmHg, and a body mass index (BMI) of 13.1 kg/m^2^.

It was revealed that she had a history of abdominal VCS, including MALS, SMAS, NCS, and MTS. Consequently, she was initiated on total parenteral nutrition (TPN), and a gastrostomy tube was inserted to facilitate nutrition intake in preparation for the scheduled intervention for her abdominal compression syndromes. However, she continued to have feeding tube dysfunction and failure to thrive. She had poor oral food intake due to post-prandial pain from complications of EDS and her various VCS. She has been in and out of various hospitals and has not had consistent nutrition since; she was unable to initiate tube feeds at home because she was not discharged with supplies or proper tube feeding education. While at the University of Maryland Rehabilitation and Orthopedic Institute, she was admitted to the University of Maryland Medical Center, given her lack of reliable nutrition for the past five to six weeks and risk for refeeding syndrome. She had a gastrojejunostomy (GJ) tube placed at the University of Maryland Medical Center due to malnourishment and failure to thrive. She was subsequently lost to follow-up, and therefore, we are unable to determine the continuity of her health status. The plan was to engage in a detailed discussion with the patient regarding the next steps. However, due to the patient being lost to follow-up, we were unable to implement this.

Despite the initial suspicion of epileptic seizures, further evaluation led to the conclusion that her symptoms were primarily attributed to syncope secondary to postural hypotension. While she was admitted to the University of Maryland Medical Center for treatment, she was originally a patient at a different hospital in Alabama. Therefore, we were unable to access her complete medical records and do not have access to any of her radiological images.

## Discussion

EDS is a heterogeneous group of connective tissue disorders characterized by varying degrees of joint hypermobility, skin hyperextensibility, and tissue fragility [3]. The genetic basis of EDS involves mutations in genes responsible for collagen synthesis and structure, leading to abnormalities in the body's connective tissues [2]. This results in a wide spectrum of clinical manifestations, from mild symptoms, such as joint pain and dislocations, to severe cardiovascular complications, such as arterial rupture [5]. The complexity of EDS makes diagnosis challenging, often requiring a multidisciplinary approach involving geneticists, rheumatologists, cardiologists, and dermatologists. The management of EDS is largely symptomatic and supportive, focusing on improving quality of life and preventing complications [6]. Physical therapy is crucial for strengthening muscles and stabilizing joints, while pain management strategies are essential for maintaining daily function [6]. In severe cases, surgical interventions may be necessary, although the fragility of connective tissues in EDS patients can complicate surgical outcomes such as hemorrhage and blood vessel rupture [5,6]. Moreover, patient education and lifestyle modifications play a significant role in managing the condition, helping individuals avoid activities that may cause injury or exacerbate symptoms. Enhanced awareness and early diagnosis are key to improving the prognosis and quality of life for individuals with EDS.

VCS represent a complex group of conditions where blood vessels or other structures are compressed by surrounding anatomical features, leading to various clinical symptoms. These syndromes, including SMAS, NCS, MTS, and MALS, present significant diagnostic and therapeutic challenges due to their overlapping symptoms and variable clinical presentations. Patients often experience chronic abdominal pain, gastrointestinal distress, and vascular complications, which can severely impact their quality of life. Moreover, studies show a strong correlation between patients with EDS and three or more types of VCS [7]. As such, a complete vascular evaluation is necessary for patients with EDS. The underlying pathophysiology typically involves abnormal anatomical relationships, such as an excessively acute angle between the superior mesenteric artery and the abdominal aorta in SMAS or the compression of the left renal vein in NCS.

Treatment strategies for VCS vary widely based on the severity and specific nature of the compression. Conservative management includes dietary modifications, nutritional support, and pain management, which can be effective in milder cases. However, for those with severe or refractory symptoms, surgical interventions become necessary [1,8]. These may range from laparoscopic procedures such as duodenojejunostomy for SMAS to more complex vascular surgeries such as stent placements or arterial revascularization in MALS [1]. However, poor outcomes have been reported in over 90% of patients who present with multiple VCS but are treated for only one of them; correction of one worsens the uncorrected vascular compression [7]. As such, correcting multiple or all VCS improves or resolves symptoms patients have with multiple VCS [7]. Advancements in imaging techniques have improved diagnostic accuracy, allowing for better visualization of vascular compressions and aiding in precise surgical planning. Moreover, minimally invasive surgical techniques have reduced recovery times and improved outcomes. Despite these advancements, the management of VCS remains challenging, necessitating ongoing research and clinical awareness to optimize patient outcomes [1].

**Figure 1:**
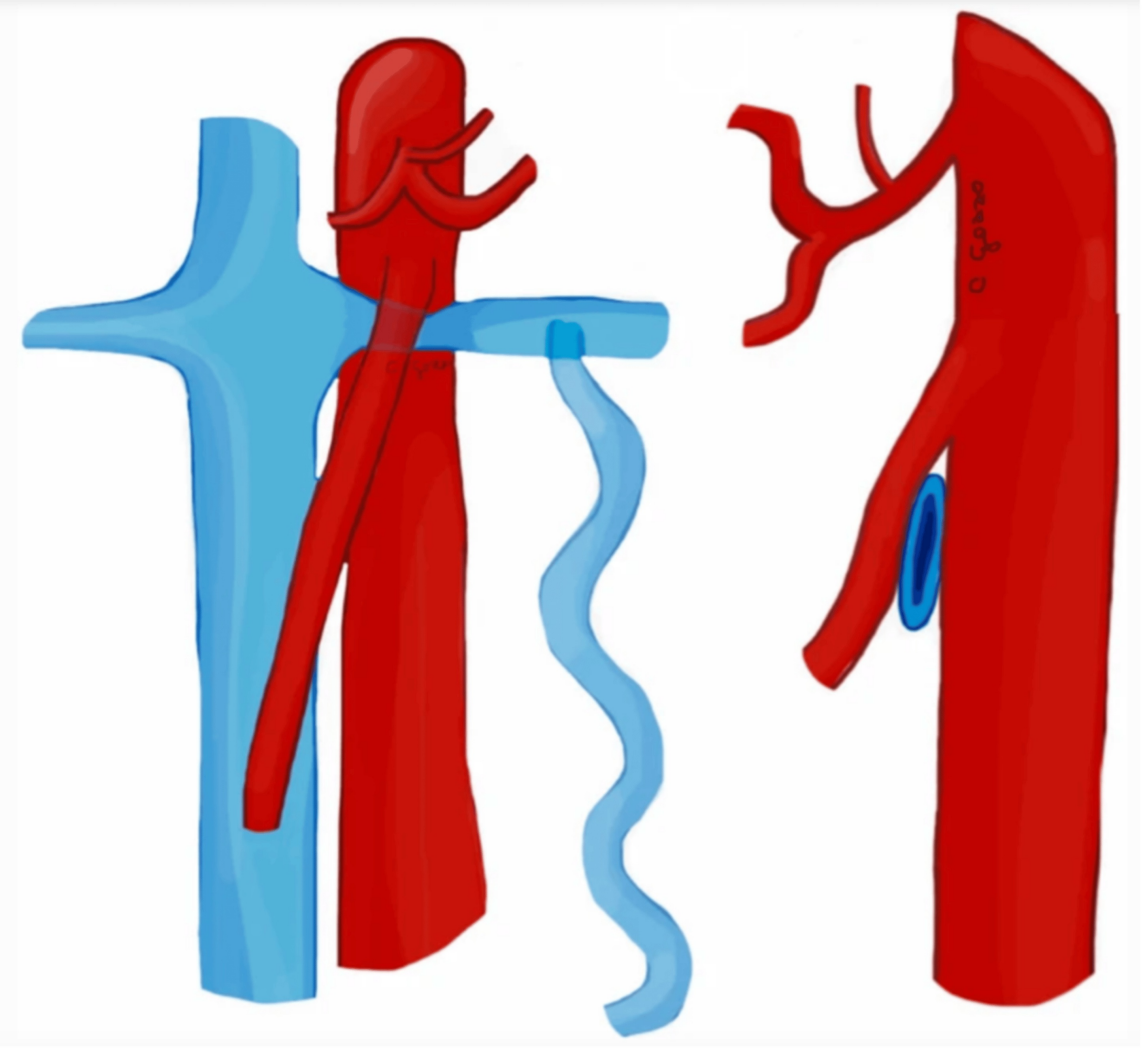
Illustration of the anterior nutcracker syndrome (NCS) in the coronal and sagittal views, respectively. The figure is taken from Gozzo et al. [1]. This article is licensed under a Creative Commons Attribution 4.0 International License, which permits use, sharing, adaptation, distribution, and reproduction in any medium or format, as long as you give appropriate credit to the original author(s) and the source (http://creativecommons.org/licenses/by/4.0/).

**Figure 2:**
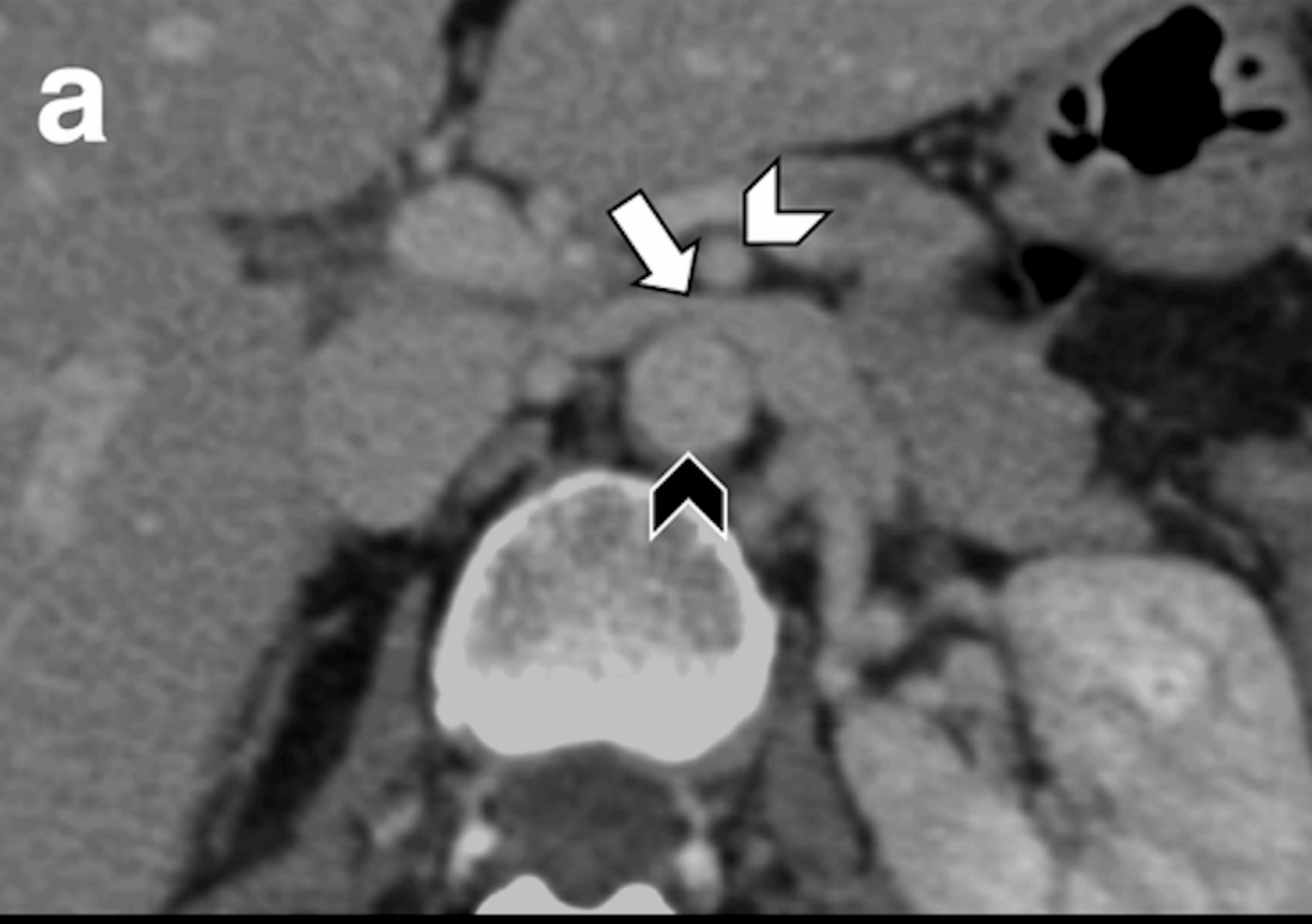
Axial view CT scan with contrast showing a “beak sign” (white arrow) indicating compression of the left renal vein between the aorta (black arrowhead) and the superior mesenteric artery (white arrowhead). The figure is taken from Gozzo et al. [1]. This article is licensed under a Creative Commons Attribution 4.0 International License, which permits use, sharing, adaptation, distribution, and reproduction in any medium or format, as long as you give appropriate credit to the original author(s) and the source (http://creativecommons.org/licenses/by/4.0/). CT: computed tomography.

NCS, also known as renal vein entrapment syndrome, is a vascular condition characterized by the compression of the left renal vein (LRV) between the superior mesenteric artery (SMA) and the abdominal aorta (Figure 1). This anatomical anomaly leads to increased venous pressure in the left kidney, resulting in symptoms such as hematuria, flank pain, and varicocele in males or pelvic congestion syndrome in females [1,3,9]. The variability in symptom severity poses significant diagnostic challenges, often requiring a combination of imaging studies, including Doppler ultrasound, CT angiography, and MRI, to confirm the diagnosis and assess the degree of compression [3,10]. A characteristic feature of NCS is the presentation of a “beak sign” (Figure 2), which indicates stenosis of the LRV between the SMA and the abdominal aorta. Management of NCS depends on the severity of symptoms and their impact on the patient's quality of life [1,3,10,11]. Conservative approaches, such as pain management and observation, are often effective and all that is needed for mild cases. However, for patients with serious or persistent symptoms such as severe hematuria and functional renal failure, more invasive interventions become necessary [3]. These surgical options can vary in terms of invasiveness, but their function is to relieve the compression and restore normal blood flow. Minimally invasive surgical methods include endovascular treatment such as renal vein stent placement [3]. Other more invasive approaches include transposition of the LRV or left gonadal vein or renal-IVC (inferior vena cava) shunt [1,10]. Despite advancements in diagnostic techniques and surgical interventions, NCS, especially in the presence of EDS, remains a complex condition with variable outcomes, necessitating ongoing research and clinical vigilance to improve patient care and prognosis.

**Figure 3:**
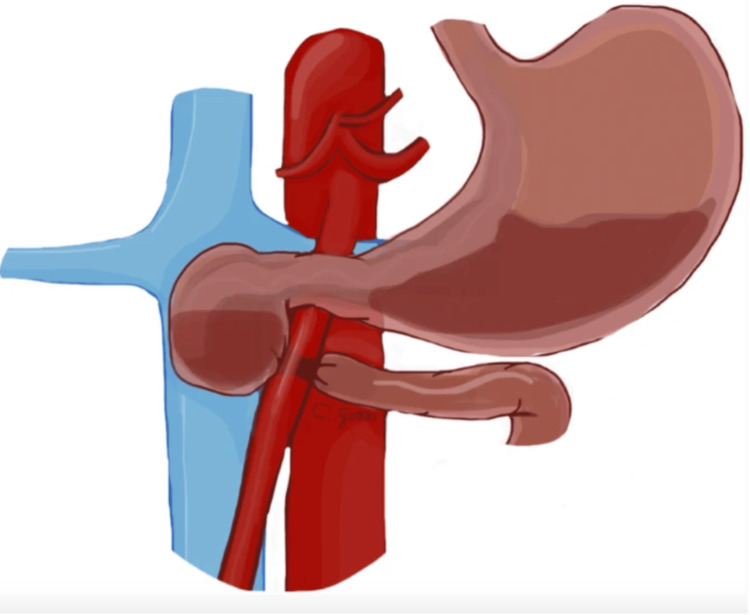
Illustration of the superior mesenteric artery syndrome in the coronal view. The figure is taken from Gozzo et al. [1]. This article is licensed under a Creative Commons Attribution 4.0 International License, which permits use, sharing, adaptation, distribution, and reproduction in any medium or format, as long as you give appropriate credit to the original author(s) and the source (http://creativecommons.org/licenses/by/4.0/).

**Figure 4:**
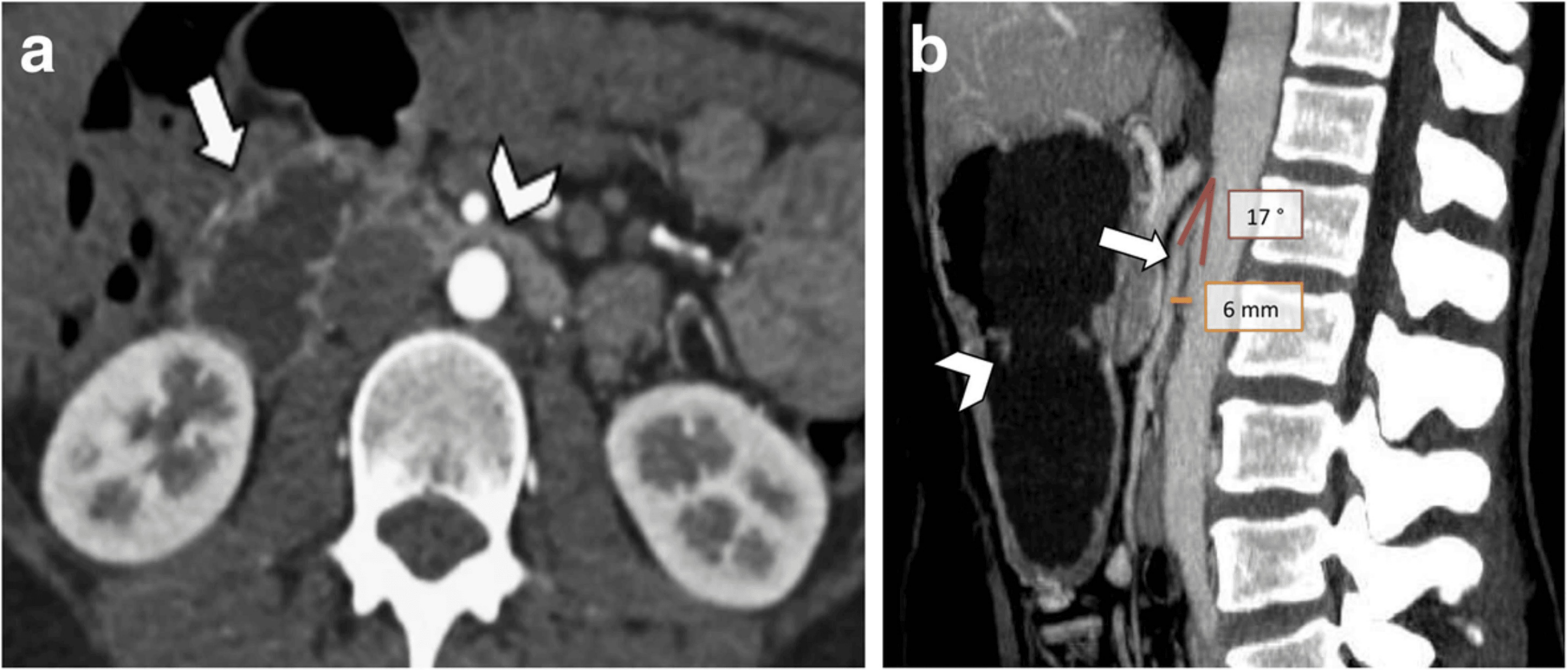
CT scan of the superior mesenteric artery syndrome. (a) Axial view CT scan showing compression of the third part of the duodenum (arrowhead) and subsequent dilation of the second part of the duodenum (white arrow). (b) Sagittal view CT scan showing the SMA (arrow), abdominal aorta, the aortomesenteric angle (17°), and the aortomesenteric distance (6 mm). Gastric dilation (arrowhead) is also present. The figure is taken from Gozzo et al. [1]. This article is licensed under a Creative Commons Attribution 4.0 International License, which permits use, sharing, adaptation, distribution, and reproduction in any medium or format, as long as you give appropriate credit to the original author(s) and the source (http://creativecommons.org/licenses/by/4.0/). CT: computed tomography; SMA: superior mesenteric artery.

SMAS, also known as Wilkie's syndrome, causes compression of the third part of the duodenum between the superior mesenteric artery and the abdominal aorta, which can cause duodenal obstruction (Figure 3) [12]. While there may not be a definitive connection between EDS and SMAS, research indicates a potential link between them [7]. The connective tissue abnormalities in EDS may contribute to the development of SMAS. This is because the laxity in the supportive connective tissues can lead to a diminished angle between the abdominal aorta and the SMA, known as the aortomesenteric angle (AMA), increasing the likelihood of compressive effects on the duodenum. A CT scan (Figure 4) is commonly used to measure the AMA in which an angle less than 22° and an aortomesenteric distance of less than 8 mm is a positive indication of SMAS [1]. Treatment options range from conservative options, with the primary objective being to increase the AMA by increasing fat stores, to surgical routes to bypass the compression. Less invasive options include increasing oral food intake to combat the weight loss associated with the condition, TPN, and decompression through a nasogastric tube [1,12,13]. Although up to 85% of patients succeed with conservative treatment, it is most effective for those whose symptoms have lasted less than a month [12]. Surgical intervention for more severe symptoms is indicated, with duodeno-jejunostomy being the procedure of choice, showing a favorable outcome in 80%-90% of cases [1,8,10,12,13]. Another surgical option includes Strong’s procedure, which involves mobilizing the duodenum to relieve the compression [8,13]. While these surgical methods can provide significant relief, careful postoperative management and monitoring are essential to ensure recovery and prevent complications.

**Figure 5:**
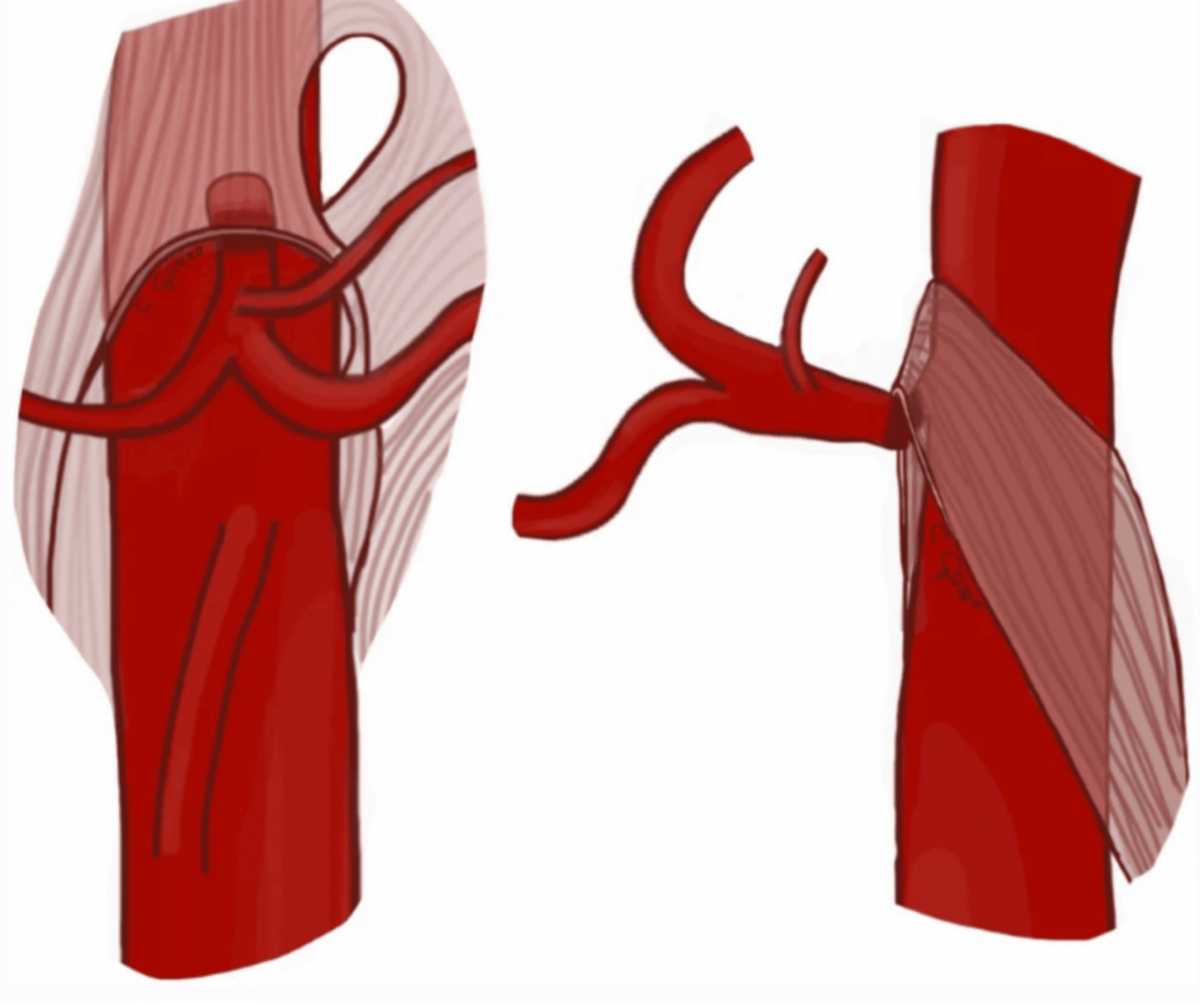
Illustration showing celiac artery compression by the median arcuate ligament in both coronal and sagittal views, respectively. The figure is taken from Gozzo et al. [1]. This article is licensed under a Creative Commons Attribution 4.0 International License, which permits use, sharing, adaptation, distribution, and reproduction in any medium or format, as long as you give appropriate credit to the original author(s) and the source (http://creativecommons.org/licenses/by/4.0/).

**Figure 6:**
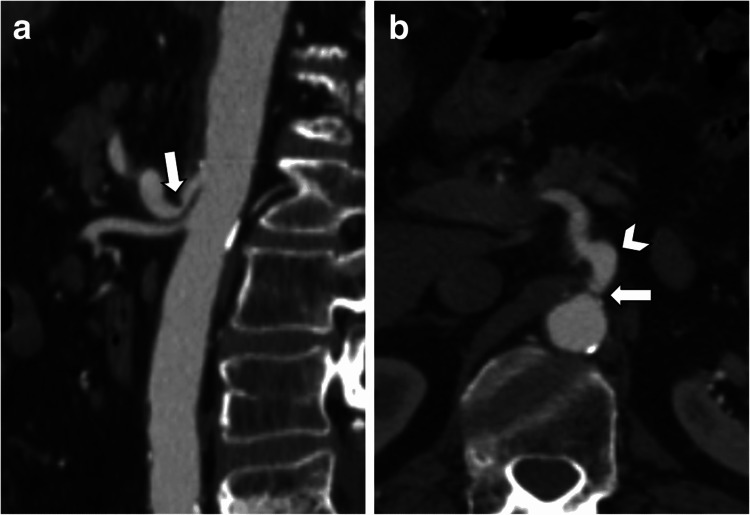
CT scan of celiac artery compression by the median arcuate ligament. (a) Sagittal view showing severe stenosis at celiac artery origin without arthrosclerosis; (b) axial view of celiac artery compression in the presence of post-stenotic dilation. The figure is taken from Gozzo et al. [1]. This article is licensed under a Creative Commons Attribution 4.0 International License, which permits use, sharing, adaptation, distribution, and reproduction in any medium or format, as long as you give appropriate credit to the original author(s) and the source (http://creativecommons.org/licenses/by/4.0/). CT: computed tomography.

MALS is another rare abdominal VCS with an incidence of 2 in 100,000 patients that is characterized by the compression of the celiac artery by the median arcuate ligament of the diaphragm (Figures 5, 6) [9]. Studies and anecdotal reports suggest a potential association between EDS and MALS [7]. Similar to the relationship proposed between EDS and SMAS, the connective tissue abnormalities inherent in EDS could contribute to the development of MALS. The laxity and fragility of connective tissues seen in EDS might lead to excessive movement or displacement of the median arcuate ligament, increasing the likelihood of compressing the celiac artery. Consequently, individuals with EDS might be at a heightened risk for MALS due to their underlying connective tissue disorder [7]. Treatment also ranges from conservative treatment to surgical release. Minimally invasive treatment entails endovascular embolization of pancreaticoduodenal aneurysm, if present, with celiac artery revascularization and stent placement [1]. Alternatively, surgical transection of the medial arcuate ligament is possible and is the method of choice [1,10]. This procedure involves cutting the median arcuate ligament to relieve the compression on the celiac artery and improve blood flow. Both methods aim to alleviate symptoms by addressing the underlying vascular compression, thereby restoring proper circulation and reducing gastrointestinal issues. These treatments are tailored to the patient's specific condition and severity of symptoms, ensuring a comprehensive approach to managing MALS.

**Figure 7:**
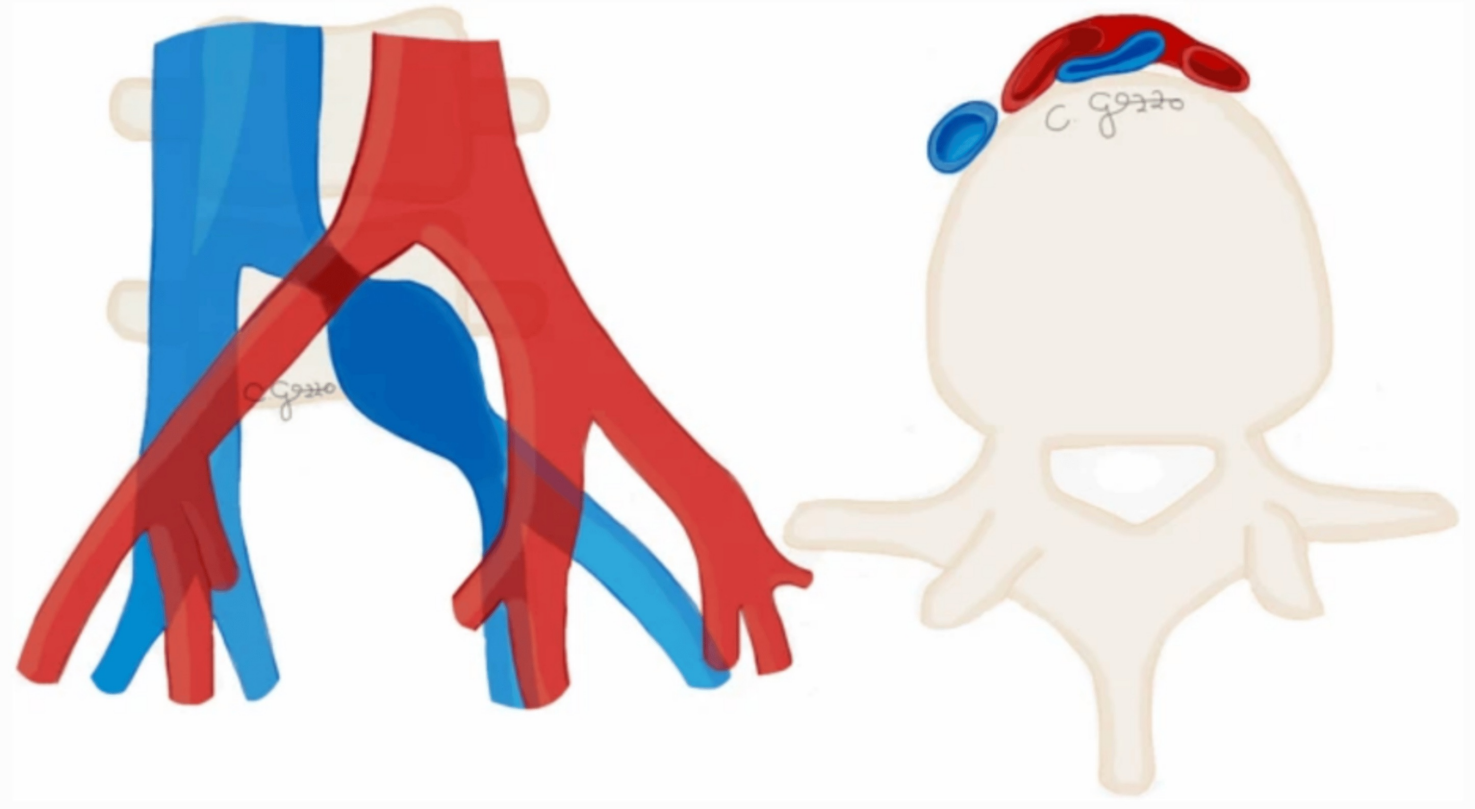
Illustration of May-Thurner syndrome (coronal and axial views). The figure is taken from Gozzo et al. [1]. This article is licensed under a Creative Commons Attribution 4.0 International License, which permits use, sharing, adaptation, distribution, and reproduction in any medium or format, as long as you give appropriate credit to the original author(s) and the source (http://creativecommons.org/licenses/by/4.0/).

**Figure 8:**
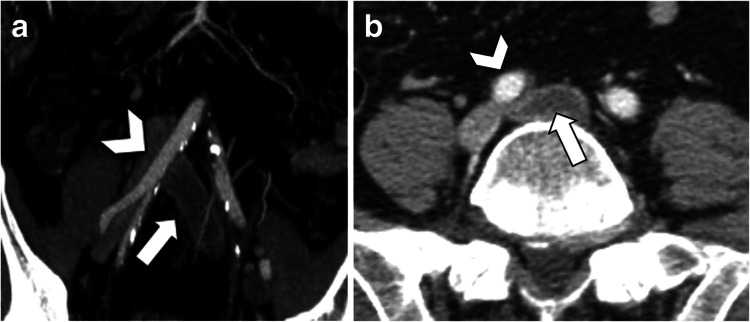
Contrast CT scan showing May-Thurner syndrome. Coronal view (a) and sagittal view (b) showing thrombosis in the compressed left common iliac vein (arrows) by the right common iliac artery (arrowheads) and the fifth lumbar vertebrae. The figure is taken from Gozzo et al. [1]. This article is licensed under a Creative Commons Attribution 4.0 International License, which permits use, sharing, adaptation, distribution, and reproduction in any medium or format, as long as you give appropriate credit to the original author(s) and the source (http://creativecommons.org/licenses/by/4.0/). CT: computed tomography.

MTS, also known as iliac vein compression syndrome, is a vascular disorder where the right common iliac artery compresses the left common iliac vein against the lumbar spine, typically at the fifth lumbar vertebrae level (Figure 7), leading to impaired venous outflow from the left leg. This anatomical anomaly predisposes individuals to deep vein thrombosis (DVT) in the left lower extremity, causing symptoms such as leg swelling, pain, and, in severe cases, venous ulcers [14]. The condition is often underdiagnosed due to its subtle presentation and the overlap of symptoms with other vascular disorders [14,15]. Diagnosis typically involves imaging techniques such as Doppler ultrasound, CT scan (Figure 8), or MRI, which help visualize the extent of the venous compression and associated thrombotic complications [14,16]. MTS management involves symptomatic relief and addressing the underlying anatomical cause [17]. Anticoagulation therapy with thrombolytics or blood thinners is the cornerstone of initial treatment to manage DVT and prevent further clot formation [14,17]. For patients with significant symptoms or recurrent thrombosis, endovascular interventions such as balloon angioplasty and stent placement are often employed to relieve the venous obstruction and restore normal blood flow [1,10,14,17]. Less commonly, more invasive surgical approaches such as thrombectomy, vascular transposition, and venous bypass may be necessary, especially when endovascular treatments are not feasible or have failed. However, they are not the preferred method of treatment due to their poor results and morbidity [1,14]. Nevertheless, most cases benefit from a combination of minimally invasive surgeries, with venoplasty being the preferred method, and clot-busting medications [17]. However, long-term follow-up is crucial to monitor for potential recurrence of symptoms and ensure the patency of the treated vessel.

## Conclusions

The coexistence of EDS with multiple VCS, including MTS, NCS, SMAS, and MALS, presents an exceptionally rare and complex clinical scenario. Each of these conditions involves distinctive anatomical anomalies leading to significant vascular and gastrointestinal complications, ranging from DVT and hematuria to bowel obstruction and chronic abdominal pain. The connective tissue abnormalities inherent in EDS exacerbate these conditions by contributing to structural laxity and fragility, complicating both diagnosis and management.

To provide comprehensive care, a multidisciplinary approach is crucial for these patients, involving geneticists, rheumatologists, gastroenterologists, vascular surgeons, and interventional radiologists. While conservative treatments may offer symptomatic relief for milder cases, severe or refractory symptoms often necessitate advanced surgical interventions such as stent placements, vein transposition, or arterial revascularization. Treating these vascular conditions concurrently can resolve symptoms and reduce poor outcomes. This complex case highlights the need for personalized treatment plans, diligent follow-ups, and increased awareness of early diagnosis and intervention, particularly in the context of EDS and VCS. Ongoing research is crucial to improving diagnostic accuracy and developing targeted therapies, ultimately enhancing patient outcomes and quality of life.
